# A Simple Way to Clear the Media for Vitrectomy in Eyes with Corneal Edema

**Published:** 2012-01

**Authors:** Touka Banaee, Alireza Eslampoor, Saeed Shokoohi Rad

**Affiliations:** Eye Research Center, Khatam-al-Anbia Hospital, Faculty of Medicine, Mashhad University of Medical Sciences, Mashhad, Iran

**Keywords:** Endothelial Cell Loss, Corneal, Silicone Oil, Keratoplasty

## Abstract

Herein we introduce a simple approach for clearing an edematous cornea during vitreoretinal surgery in eyes with decompensated corneal endothelium, allowing the surgeon to postpone penetrating keratoplasty. This technique was performed in 3 eyes by filling the anterior chambers with air or silicone oil, and sufficiently cleared the media for completion of vitrectomy. This simple technique enables completion of the vitrectomy without a temporary keratoprosthesis and penetrating keratoplasty in eyes with corneal edema due to endothelial decompensation.

## INTRODUCTION

There are instances when a vitreoretinal surgeon faces retinal detachment or other posterior segment complications requiring vitrectomy, yet without adequate media clarity as a result of corneal endothelial decompensation. In these circumstances, the conventional approach is to proceed with a temporary keratoprosthesis followed by penetrating keratoplasty at the end of the surgery after finalizing the vitreoretinal procedure. It is commonly believed that graft survival is less favourable in silicone oil-filled eyes.^1^,^2^ One logical approach would be to find a way for clearing the cornea intraoperatively for the sake of the vitrectomy and delay penetrating keratoplasty until later removal of silicone oil. There are also times when the cornea becomes edematous intraoperatively hampering detailed surgery. What should the surgeon do in such cases?

## SURGICAL TECHNIQUE

There is a simple solution to this problem. By filling the anterior chamber with a clear substance such as air, silicone oil or ophthalmic viscosurgical devices, which in contrast to fluids do not penetrate the cornea, one can make the cornea clear enough to conclude the surgery.

## DISCUSSION

Vitreoretinal surgeons encounter many patients with silicone filled anterior chambers where the oil covers the entire posterior corneal surface. Surprisingly, these eyes often have clear corneas despite presumed damage to corneal endothelial cells. We assume such corneas owe their clarity to the fact that when the endothelium is covered with silicone oil, there is no access for fluid to enter the cornea, therefore the cornea remains clear. This may be due to the principle “no water, no edema”, no matter how the endothelium functions. The same principle may also be employed during surgery on eyes with corneal edema. When the edema is significant enough to preclude adequate visualization during surgery, one may inject air or silicone oil into the anterior chamber to make the cornea clear. In three vitrectomy cases of ours, we encountered such a problem and applied air (one eye) or silicone oil (two eyes) injections for this purpose; the cornea became clear within a few minutes and surgery was continued with no difficulty. Descemet’s folds occurred more readily under air.

**Figure 1.**
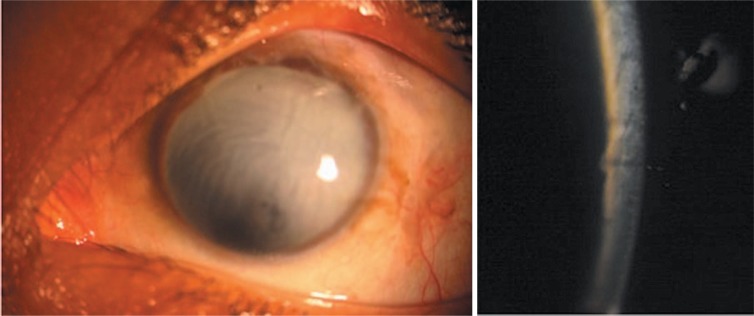
Completely opaque cornea (left image) due to the presence of a retrocorneal membrane (right image).

**Figure 2.**
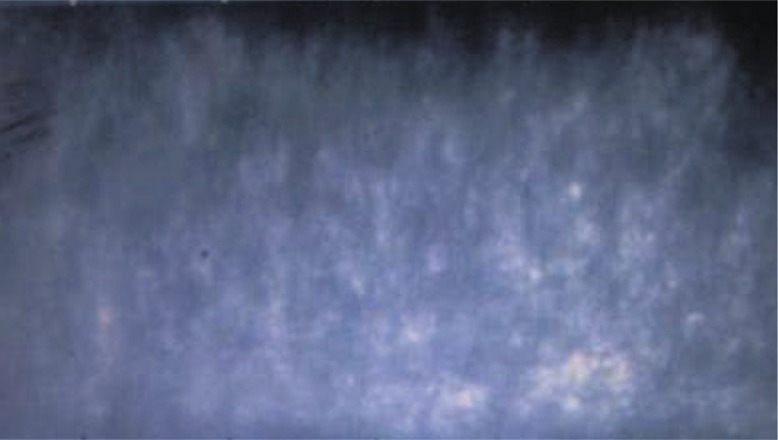
Specular microscopy of the same eye as in figure 1, showing the absence of corneal endothelial cells.

**Figure 3.**
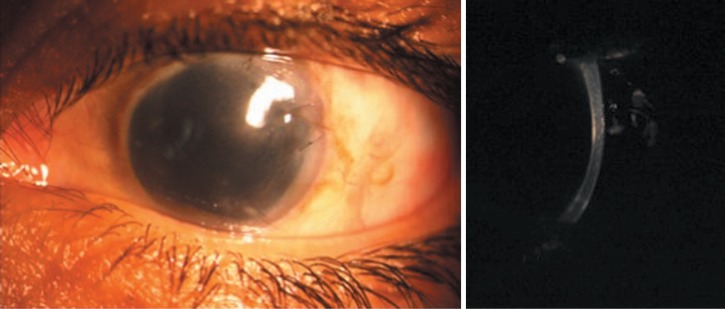
Same eye 2 weeks after removal of the retrocorneal membrane. The cornea has cleared to a large extent (left image) without edema (right image). Remnants of the membrane are visible peripherally (arrow).

To better demonstrate the principle of “no water, no edema”, we present a patient with multiple previous vitreoretinal operations, an opaque cornea with fibrotic retrocorneal membrane (Fig. 1) devoid of endothelial cells as demonstrated by specular microscopy (Fig. 2). The retrocorneal membrane was removed during vitreoretinal surgery and 2 weeks afterwards the cornea which was in direct contact with silicone oil, became clear despite the absence of endothelial cells (Fig. 3).

One may argue that the endothelium may sustain additional damage by the injection of air or silicone oil. The response would be that, a damaged endothelium is better than a damaged retina in an eye which the need for penetrating keratoplasty is imminent. Viscosurgical devices are another alternative for this purpose which may avoid additional damage to the corneal endothelium.
